# Attractor States in Teaching and Learning Processes: A Study of Out-of-School Science Education

**DOI:** 10.3389/fpsyg.2017.00299

**Published:** 2017-03-03

**Authors:** Carla H. Geveke, Henderien W. Steenbeek, Jeannette M. Doornenbal, Paul L. C. Van Geert

**Affiliations:** ^1^Department of Teacher Education and Center of Expertise Healthy Ageing, Hanze University of Applied SciencesGroningen, Netherlands; ^2^Department of Developmental Psychology, Faculty of Behavioural and Social Sciences, University of GroningenGroningen, Netherlands

**Keywords:** expressed pedagogical content knowledge, teaching and learning processes, complex dynamic systems approach, attractor states, out-of-school science education

## Abstract

In order for out-of-school science activities that take place during school hours but outside the school context to be successful, instructors must have sufficient pedagogical content knowledge (PCK) to guarantee high-quality teaching and learning. We argue that PCK is a quality of the instructor-pupil system that is constructed in real-time interaction. When PCK is evident in real-time interaction, we define it as Expressed Pedagogical Content Knowledge (EPCK). The aim of this study is to empirically explore whether EPCK shows a systematic pattern of variation, and if so whether the pattern occurs in recurrent and temporary stable attractor states as predicted in the complex dynamic systems theory. This study concerned nine out-of-school activities in which pupils of upper primary school classes participated. A multivariate coding scheme was used to capture EPCK in real time. A principal component analysis of the time series of all the variables reduced the number of components. A cluster revealed general descriptions of the components across all cases. Cluster analyses of individual cases divided the time series into sequences, revealing High-, Low-, and Non-EPCK states. High-EPCK attractor states emerged at particular moments during activities, rather than being present all the time. Such High-EPCK attractor states were only found in a few cases, namely those where the pupils were prepared for the visit and the instructors were trained.

## Introduction

### A microgenetic view on pedagogical content knowledge in out-of-school teaching and learning

Out-of-school environments, such as museums, parks, science centers, planetariums, zoos, are highly valued resources for learning about science (Griffin, 2004). In such environments, science education usually occurs under the guidance of an instructor (Salmi, 2012) and comprises activities that are authentic, realistic, meaningful and free to choose. As formal learning in school and less formal learning in out-of-school facilities take place in different contexts, they show differences as well as similarities. Tran (2006), for instance, showed that the educational goals of museum instructors are more focused on pupils' interest and excitement than on the aspect of cognitive learning, in contrast with teachers, whose educational goals are to a considerable extent determined by the fact that, as teachers, they are accountable for academic learning. Kisiel (2010), on the other hand, states that the informal out-of-school educational practices are comparable with formal educational practices, and that they both involve for instance presentations, demonstrations and hands-on activities. However, the pedagogy of out-of-school science practices, e.g., ways of giving instruction and how they relate to pupils' level of cognitive learning, has scantly been investigated.

In school contexts, an important indicator of pedagogical quality of science education is the teachers' Pedagogical Content Knowledge (PCK). Shulman (1986, p. 9) defined teacher's PCK as “Ways of representing and formulating the subject [content] that make it comprehensible to others…this also includes an understanding of what makes the learning of specific topics easy or difficult.” High-quality practices (as defined by PCK) in out-of-school learning are likely to be comparable with those in formal in-school teaching, considering the fact that instructors and teachers face the same challenge of teaching pupils about science. In formal learning situations, PCK is composed by teachers' knowledge of and beliefs about goals for teaching, the curriculum, instructional strategies, pupils' understanding of specific topics, general pedagogy, and different contexts (Coenders, 2010; Platteel, 2010).

However, it is not enough for teachers to have this knowledge, it must also be expressed in their concrete teaching activities and interactions with pupils (Henze and Van Driel, 2015; Park and Suh, 2015). Although, Park and Oliver (2007) argue that components of PCK are intertwined and develop during (inter)action in the classroom, they are considered qualities of the teacher. In this article we argue that the expression of PCK is first and foremost a *dynamically emerging* phenomenon that is *co-constructed* during *real-time interaction* between teacher and pupils. EPCK is thus a quality of the teacher-pupil system. We call this emergent process of mutually influencing components Expressed Pedagogical Content Knowledge (EPCK).

To investigate how EPCK emerges through co-construction, we have to observe the process as when and where it actually occurs (Schauble et al., 1997). This asks for a microgenetic approach (Flynn et al., 2007), which means that the process is observed in an authentic context—the actual out-of-school environment—and in real time, i.e., by observing the process as it unfolds over seconds and minutes during real teacher-pupil interaction.

If EPCK is constructed in real time by teachers and pupils and defined by intertwined components, research into the emergence of EPCK could profit greatly from a complex dynamic systems approach. However, studies on PCK have thus far not taken into account the complex dynamic systems (CDS) point of view and PCK has merely been investigated in formal learning contexts (in schools, as a “background quality” of the teacher). In this study we present an alternative way of exploring EPCK: adopting a microgenetic approach (Flynn et al., 2007) that is inspired by a CDS point of view and applying it to out-of-school science education.

### Principles of complex dynamic systems: basic concepts

The teaching and learning process is a socially situated, complex system that is composed of specific, and dynamically evolving combinations of multiple elements or variables (Kunnen and Van Geert, 2011; Steenbeek and Van Geert, 2013). Examples of such elements or variables are the teacher's and the pupils' verbal and nonverbal actions, emotional evaluations, memory and so forth. These elements interact: for instance, a pupil may verbally react on a question posed by the teacher, and the teacher may react on the pupil's answer, whereas other pupils may react in the form of thoughts or feelings on this particular pupil-teacher interaction. It is clear that the network of interactions is extremely complex and changing all the time (hence complex dynamic system) (Vallacher et al., 2010; Kunnen and Van Geert, 2011). In order to conceptualize and describe a particular complex network of many connected elements or variables, researchers focus on a small part of the network (Kunnen and Van Geert, 2011). This may be a collection of three or more characteristic variables, which form a descriptive reduction of the complex system. According to CDS theory, the behavior of the entire complex system is mapped onto this small set of descriptive variables, and important properties of the complex system may be understood by studying its behavior over time in this highly reduced descriptive model. The set of variables used to describe a complex system may be conceived of as dimensions of a space, for instance a three-dimensional space if three descriptive variables are used. Each point in the space corresponds with a value on each of the descriptive variables, and can thus be interpreted as a possible or observed state of the system in this space. This descriptive space is also called the state space (or sometimes phase space) of the complex system. A helpful metaphor is to conceive of the complex system as being concentrated in the form of a fly, and the state space as consisting of three spatial dimensions, describing a room. The fly (the complex system) may fly around in a completely erratic way, with no preference for a particular place in the room. Or, alternatively, the position of the fly in the room may be entirely determined by external forces (e.g., it may stick to a flycatcher). However, an important characteristic of a complex system is that it tends to spontaneously evolve toward a particular state (fly to a particular place in the room) and stay there, while resisting external attempts to be moved (if chased away, the fly will tend to fly back to this particular place). This place or region in the state space toward which the system spontaneously evolves and which it actively sustains, at least for a while, is called an attractor state. For instance, an educator's questions that focus on insights elicit pupils' conceptual understanding, which in turn makes it easier for the educator to ask high-level questions. This typically forms a self-sustaining cycle of a certain duration.

A complex system may have one such region to which it spontaneously evolves (one attractor) or there may be several such states, each of which has its characteristic features (multiple attractors; e.g., the fly has various places in the room to which it tends to fly and stay for a while; Hollenstein, 2007; Kunnen and Van Geert, 2011). The system may move from one attractor to another on the basis of internal drives, and/or on the basis of some external force (which is then called a perturbation). The fact that there is a limited number of such states implies that there exists a certain repetitiveness or recursiveness in the states visited by the system. A complex system can be characterized by the properties of its attractors and by the characteristic way it moves toward or away from particular attractors, e.g., does it move quickly from one attractor to another, or is the pattern between two attractors erratic and thus long-lasting, is it easily driven out of a particular attractor by some perturbation or does it resist such forces, etc.; (Kunnen and Van Geert, 2011; De Ruiter et al., 2017).

### Components and variables describing the state space of EPCK

In science education, PCK is traditionally defined by means of its components, namely: teachers' orientation toward science (e.g., goals and objectives of teaching) and their knowledge of and beliefs regarding the science curriculum, pupils' understanding of science, assessment in science, and instructional strategies for teaching science (Magnusson et al., 1999; Espinosa-Bueno et al., 2011; Friedrichsen et al., 2011; Chapoo et al., 2014; Henze and Van Driel, 2015; Park and Suh, 2015). In most studies, PCK is seen as solely consisting of internal components, i.e., representations a teacher's knowledge and beliefs (Baxter and Lederman, 1999). Although, recently some scholars have acknowledged the importance of studying the enactment of PCK in the classroom (see studies of e.g., Henze and Van Driel, 2015; Park and Suh, 2015), more knowledge is needed of how PCK components are expressed during real-time interaction. Additionally, PCK is defined as a quality of the teacher, whereas EPCK is considered to be a process of intertwined interactions that take place between teacher and pupils in a particular context.

Based on a variety of studies (e.g., Rowe, 1974; Van Zee and Minstrell, 1997; Chin, 2006; Oliveira, 2010; Alfieri et al., 2011; Engel, 2011; Haug, 2014), we defined seven High-EPCK components as those that reveal relevant, observable aspects of high-quality science teaching and learning processes. The central component for High EPCK is the *Allocated learning time* (Stallings, 1980)—the time that is actually spent on the content and which is conditional for the presence of the other six components. These other components can be divided into educator components (components that refer to teachers or instructors) and pupil components. The first educator component for high-level EPCK is *Open teaching style focused on conceptual understanding*. This component is related to the key PCK component *Knowledge of instructional strategies* (Park and Suh, 2015). Studies show the importance of asking questions that provoke pupils' thinking (Oliveira, 2010; Wetzels, 2015), using minimal verbal and nonverbal responses to encourage pupils to speak (Cazden, 2001; Sawyer, 2006), and of providing think-time (e.g., Rowe, 1974). Also essential for high-quality teaching and learning is an open teaching style that is focused on conceptual understanding, rather than on declarative knowledge. The second educator component for High EPCK is *Reaction to pupil's contribution*. This component is related to key PCK component *Knowledge of pupils' understanding* (Park and Suh, 2015). Through giving adequate responses to pupils' contributions, the educator accepts that they may actively participate in the conversation and thereby supports pupils' cognitive autonomy (Stefanou et al., 2004). If spontaneous contribution of pupils is promoted, authentic learning situations may be created in which genuine understanding emerges due to the pupils' commitment to learning and experienced ownership (Iran-Nejad and Stewart, 2010). The third educator component is *Reaction to pupil's conception*. This component is related to key PCK component Knowledge of pupils' understanding (Park and Suh, 2015). In high-quality teaching and learning, the educator responds in a neutral way to the content of pupils' contributions by avoiding an overly judgmental attitude (Van Zee and Minstrell, 1997) and by asking follow-up questions to encourage pupils to further explore the subject.

The pupil components are strongly related to the educator components. The first pupil component important for high-level EPCK is *Conceptual understanding*. Conceptual understanding is a deeper form of knowledge than declarative, factual knowledge. The second pupil component is *Pupil's contribution* to the conversation. When pupils are invited to contribute to the interaction, it increases their intrinsic motivation to learn about science (Berlyne, 1960; Csikszentmihalyi and Csikszentmihalyi, 1988) and stimulates their cognitive autonomy and self-regulation (Stefanou et al., 2013). The third pupil component is *Pupil's conception*, which can be correct, fragmented or incorrect according to accepted scientific ideas. If pupils express their concepts, it enables educators to adapt their education to the needs of those pupils.

### Investigating EPCK as a complex dynamic teaching and learning system

In this study we suggest that, in order find and describe states of EPCK—i.e., the EPCK attractors if any such exist—the micro-interaction dynamics between variables within and across EPCK components should be studied, using the properties of how complex dynamic systems move through their state space (Van Geert, 1994; Smith and Thelen, 2003; Kupers, 2014). The first of these CDS properties is that EPCK is composed of iterations of interconnected “teaching variables” of educators and “learning variables” of pupils. Iteration implies that a preceding state forms a condition for the next state, thus forming a conditionally connected sequence of states. This sequence of states corresponds with the systems' trajectory through the state space. It is this trajectory, i.e., this particular process in time that has to be studied in order to understand the nature of the complex dynamic system. These iterations show reciprocal causality over time (Guanglu, 2012)—for example, educator's questions elicit conceptual understanding in pupils in this conceptual understanding elicits further questioning. This iterative, reciprocal causality leads to the emergence of particular EPCK attractor states. That is such states are the product of self-organization, as opposed to merely being the sum of the contributions of independent variables (cf. Den Hartigh, 2015).

The second CDS property is the interconnectedness of different timescales, in particular the short-term timescale of pupils interaction in real-time, and the long-term timescale of changes in the pattern of such interactions (e.g., the pattern and properties of attractors; Steenbeek and Van Geert, 2013). The real-time interactions that take place during an out-of-school activity influences the long-term development, but is also a result of the long-term development. To obtain a deeper understanding of the complex dynamic system, it is necessary to repeatedly observe sequences of real-time teaching and learning processes and check whether the dynamic patterns of teaching and learning change when lessons or activities are repeated over a longer period of time.

The third property teaching and learning processes as a CDS is that developmental processes show non-linearity and variability over time. The system's short and long-term trajectories cannot be represented as smoothly and gradually changing lines, but instead show a characteristic pattern of variability and fluctuation (Kupers, 2014). For instance, variability may temporarily increase and this increase can provide an indication that the teaching and learning process is about to change, for instance that it is moving toward higher or lower levels of EPCK (e.g., Bassano and Van Geert, 2007).

**Figure 1:**
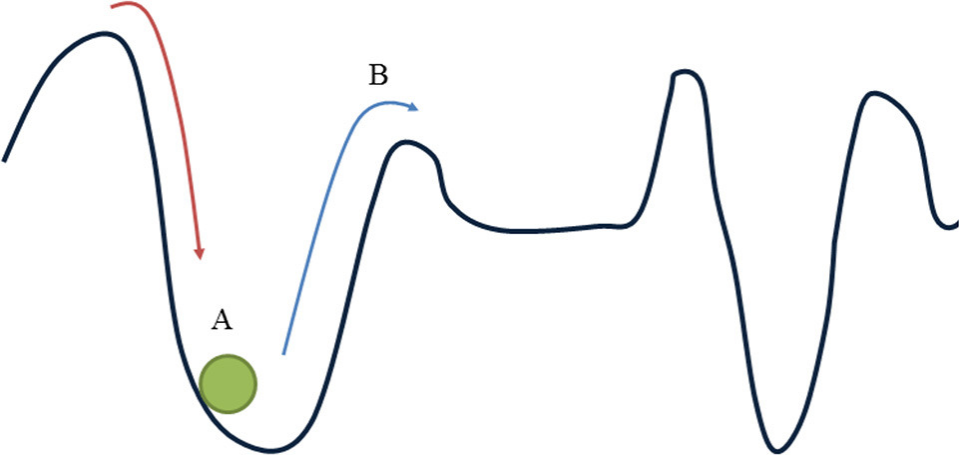
**Attractor landscape**. Theoretical model of possible conditions of the teaching and learning and learning system at a certain moment in time.

The fourth CDS property is the forming of attractor states, which have been explained already in section Principles of Complex Dynamic Systems: Basic Concepts. In principle, an attractor state is a temporarily self-sustaining state. According to various authors (Meindertsma, 2014; De Ruiter et al., 2017), indications for attractor states can already be observed on a short-term timescale, based on the pattern of short-term variability of the elements or variables. All possible states of the system can be represented by means of an EPCK attractor landscape. Figure 1 is a representation of such an attractor landscape. The classical metaphor that is used to help understand the notion of attractor landscape is that of a landscape of hills and valleys, and a ball—subjected to the laws of gravity—rolling across the landscape, with the current position of the ball corresponding with the current state of the system (A). This metaphor (which now reduces the state space to two dimensions, left right and down) aims to illustrate all possible conditions of the system at a certain moment in time. Valleys metaphorically correspond with attractors, since the ball tends to roll down the valley and comes to a halt when it reaches the bottom (that is, the system as it reached its temporarily stable, self-sustaining attractor state). The system may move out of the valley as a consequence of internal forces, or as a consequence of external forces, namely perturbations that tend to push the ball out of the valley (B). The deeper the valley, the more external force must be exerted to push the ball over the rim. The depth of the valley thus corresponds with the strength and self-sustaining force of a particular attractor (for applications to educational systems, see for instance Meindertsma, 2014). If there are no EPCK attractor states, the attractor landscape tends to be flat, which means that the system will follow an arbitrary path in the state space, due to accidental, mainly external influences. Instead of a self-organizing pattern it shows a variable pattern without a stable structure, which implies a constantly changing teaching and learning process.

**Figure 2:**
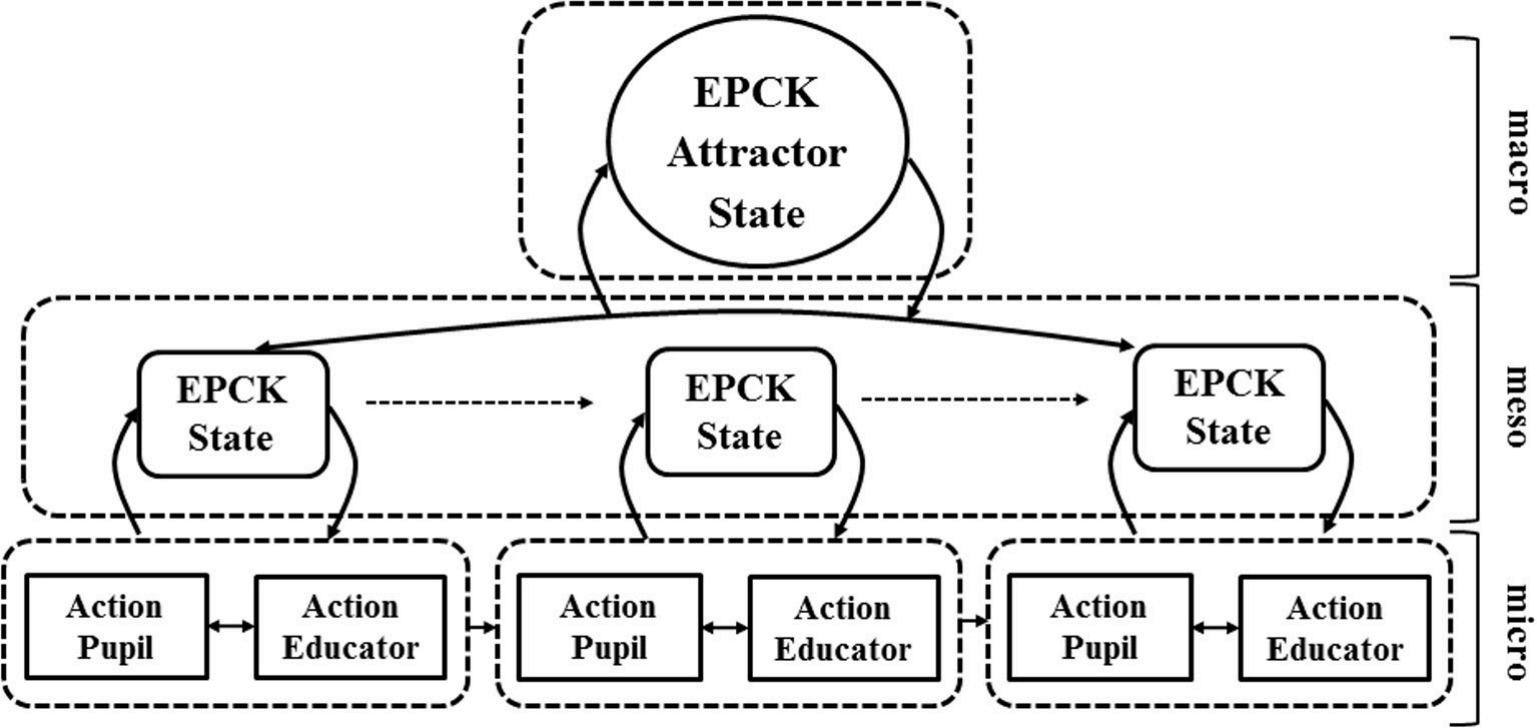
**Model of self-organizing expressed pedagogical content knowledge**. High-quality teaching and learning interactions at the micro level, which self-organize into sequences of high-level conversation at the meso level, and finally stabilize into attractor states of high-quality teaching and learning at the macro level.

In a different field of investigation—namely that of trait and state self-esteem—De Ruiter et al. (2017) has presented a model of how attractor states self-organize on a variety of timescales. De Ruiter et al. (2017) describes self-organization of self-esteem states on the meso level of parent-child interaction (typical duration: a matter of minutes), composed of a constellation of coherent elements (variables, such as self-directed feelings and thoughts) on the micro level (typical duration: a matter of seconds). The waxing and waning of these meso-level states determines the long-term development of self-esteem on the macro level. At the same time, the state of long-term development of self-esteem determines the self-assembly of micro- and meso-level patterns. This pattern of dynamic relationships between self-organizing processes on three distinct timescales can also be applied to of EPCK attractor states. On the macro level, the emergence of EPCK attractors, is likely to be determined by real-time teaching and learning states, that is, states occurring on the meso-level timescale. These meso-level states are influenced by the specific combination of actions of pupils and educators on the micro-level timescale. If on the micro level the interaction is a self-sustaining state composed by high-quality teaching and learning variables (such as conceptual understanding, asking questions, providing think-time) and if these variables self-organize into a high-quality conversation on the meso level (minutes), the state can be characterized as a High-EPCK state. If the state's duration is substantial, shows low variability—both relative to other potential states in the teacher-pupils interaction—and if the state occurs repetitively, i.e., in a recurrent pattern, the state qualifies as an attractor state in the interaction dynamics of this particular teacher and pupils (see Figure 2). Unfortunately, long-term attractor states, i.e., states that show a recurrent pattern across several lessons, are difficult to find in out-of-school activities, as they often amount to a single visit. In this study, macro level attractor states are based upon the recurrence and duration of these states across the total length of the observation during one out-of-school visit.

### Idiosyncrasy and commonality of EPCK in out-of-school activities

In general, learning is the result of constant feedback loops that take place during teacher-pupil interaction on the micro level (Howe and Lewis, 2005). Such feedback loops, and the resulting emergence of (attractor) states on the meso and macro level, tend to occur in the form of relatively idiosyncratic processes. In spite of this idiosyncrasy and of the idiosyncratic nature of the emerging attractors, we can expect to find similarities across teacher-pupils systems and across contexts (e.g., Kupers, 2014). It is likely that this idiosyncratic nature is particularly salient in out-of-school activities, due to the complex, variable and less structured nature of these environments (Crowley et al., 2000; Bamberger and Tal, 2008). If teachers have prepared their pupils in the classroom prior to the visit for the visit teaching and learning patterns in the out-of-school activity are likely to be less idiosyncratic, as prior knowledge pre-structures the out-of-school activity and the information it presents. However, even in the case of prepared activities, idiosyncrasy is plausible whenever pupils have been given a large amount of cognitive autonomy.

As far as the teacher is concerned, literature shows that there are considerable individual differences between teachers' PCK (Van Driel et al., 1998; Coenders, 2010). Even when teaching concerns the same subject matter, large individual differences have been found (Park and Oliver, 2007). This might also be the case for instructors in out-of-school activities. In spite of the many differences, researchers also found characteristics of PCK that were common between teachers (Loughran et al., 2004; Park and Oliver, 2007). A common characteristic of High EPCK, for instance, is the nature of teachers' questions to enhance pupils' conceptual understanding.

Another way to reduce the idiosyncrasy of out of school teaching-learning patterns is to provide training to instructors, aimed at facilitating high-quality science teaching that is focused on conceptual understanding (Wetzels, 2015; Geveke et al., 2016). However, even if teachers have undergone the same teacher training, they might perceive the training in different ways and therefore apply what they have learned differently (Van Driel et al., 1998; Wetzels, 2015). In conclusion, idiosyncrasy must be taken into account when investigating EPCK.

### Questions and hypotheses of the present study

In this study we argue—on theoretical grounds—that indications of the emergence of high-quality practices consisting of High EPCK can be observed in the short-term micro-interaction dynamics within out-of-school science activities. The aim of this study is to empirically explore the real-time teaching and learning process in various out-of-school science activities, to examine whether and how the teaching and learning processes self-organize into attractor states, and to determine the characteristics of these states in relation to the different out-of-school activities. Although, many researchers assert that the development of PCK is an active and dynamic process (e.g., Van Driel et al., 1998; Park and Oliver, 2007; Henze et al., 2008; Coenders, 2010), it is generally not approached as such. This study fills a void in the existing literature by using the dynamic systems approach to empirically investigate the emergence of the expressed form of PCK during out-of-school activities. As this study only includes “one-off” visits to out-of-school facilities (a visit is usually a one-time event), we were not able to study EPCK on the long-term timescale (second CDS property).

Our first question that concerns the empirical exploration of the emergence of EPCK on the micro level timescale is *Can multiple teaching and learning variables be reduced to a limited number of underlying teaching and learning components, and if so, are these underlying components idiosyncratic or general across different instructors and out-of-school activities?* As the intertwined teaching and learning process is composed of a large number of interacting teaching and learning variables, it is hard to conceptualize the interconnected network without reducing it to a much smaller number of underlying components or variables. We are interested in finding out how the underlying variables are interconnected in terms of their time-serial structure within and across components during particular teaching-learning processes. We expect that the teaching and learning components will be idiosyncratic (unique combinations of elements or variables) but will also show similarities in terms of general comparability (resemblances in terms of combinations of particular variables).

Our second question focuses on how the intertwined teaching and learning process self-organizes into meso-level states: *How do successive moments in an actual out-of-school activity cluster into qualitatively distinct successive teaching and learning states, and how comparable are the self-organizing processes of these states across different cases?* Our expectation is that if we find teaching and learning components at the micro level, these components will self-organize into (potential attractor) states at the meso level. We also expect that, although these states are by definition idiosyncratic, we will find content-related similarities, that is, that the same components are active in different cases.

Our third question aims to compare the cases in their stabilizing patterns on the macro level: *What are the characteristics of the attractor states, if any are found, and can the data be related to the properties of different out-of-school activities?* On the short-term timescale of one out-of-school activity, we expect to find attractor states on the basis of three criteria, namely recurrent pattern, duration, and low variability of the self-assembly of components into states. We expect that out-of-school activities with similar properties (topic and organization), for which pupils have been prepared, and with instructors who are trained at high-quality teaching (using an open teaching style focused on conceptual understanding) might nevertheless show different EPCK attractors states, due to the idiosyncratic nature of the teaching and learning processes. However, we also expect to find similarities between attractor states.

## Methods

### Subjects of the study

This study was approved by the Ethical Committee Psychology of the University of Groningen (the Netherlands). Instructors and the parents of the pupils filled in an informed consent form before this study.

This study contains nine cases of primary school classes taking part in different out-of-school activities. The participants consist of pupils of upper primary school classes of a variety of schools in the north of the Netherlands (grade 3–6). All of the schools were connected to a program of the Northern Netherlands Science Network and had agreed to participate in the networks' research into out-of-school science activities. The program consisted of at least one visit to an out-of-school activity. The activities included in this study are a visit to: the Kapteyn Mobile Planetarium, the University Museum, Children's University, the Science Center, and the Mobile Science Classroom (a truck called the Salt Express). The Science Network offered preparation programs for the activities, although not all schools prepared the pupils before taking part in visiting the out-of-school activity (Table 1). The instructors included in this study were asked to follow a half day's training session, which half of the instructors featured in this study had done. The training concerned information about open teaching, focused on eliciting conceptual understanding. The cases described in this study were chosen from a larger set of recorded out-of-school activities. This selection was based firstly on whether the interactions in the recorded activities showed a level of variability that sufficed to discriminate between various kinds of patterns during a single recorded activity or during a single session, and secondly on whether it was likely that some of these patterns would comply with our definition of High EPCK. Table 1 lists the characteristics of the selected cases.

**Table 1:** **Overview of the properties of the cases**.

**Cases**	**Activity description**	**Pupils Grade and preparation**	**Instructor Gender and training**
Case 1	Mobile Planetarium, interactive presentation, 17 pupils	Grade 5–6, prepared	Male, trained
Case 2	Science center, inquiry learning, 2–3 pupils	Grade 5–6, prepared	Female, untrained
Case 3	Children's University, lecture presentation, 200 pupils	Grade 5–6, semi-prepared^a^	Female, untrained
Case 4	Science Center, inquiry learning, 4 pupils	Grade 5, prepared	Female, trained
Case 5	Mobile Science Classroom, interactive presentation, 17 pupils	Grade 4–6, unprepared	Male, untrained
Case 6	Mobile Planetarium, interactive presentation, 24 pupils	Grade 6, unprepared	Female, trained
Case 7	University Museum, inquiry learning, 2–5 pupils	Grade 4, unprepared	Male, untrained
Case 8	Mobile Science Classroom, interactive presentation, 17 pupils	Grade 4–6, prepared	Male, trained
Case 9	Children's University, lecture presentation, 150 pupils	Grade 5–6, unprepared	Male, untrained

*Dutch version of a presentation of the training can be requested from the author*.

a *Pupils of different schools; not all participating classes were prepared for the lecture*.

### Procedure

The video observations we carried out did not interfere with the natural educational context, meaning neither the instructor or the pupils were prescribed to behave in a certain way, follow a certain procedure, or apply a particular teaching style. The duration of the activities differed in each case and ranged from 20 min to 1.5 h.

In order to code EPCK, a coding scheme with 26 variables was used (see Table 2). The coding system entailed variables referring to High-EPCK content, and variables referring to non-EPCK interactions. We coded each variable with 0 or 1 for each precise moment, using the video coding system Mediacoder (Bos and Steenbeek, 2010) to capture the presence of each variable at any time. In Mediacoder, media files can be imported; behavior can be coded exactly on time, which means the codes can directly be retraced in the media files. The coded data can be exported into Excel the carry out further analyses. We tested the optimal coding length for capturing the dynamics of the main variables, and this resulted in 600 s of coding for each case.

**Table 2:** **EPCK coding scheme with theoretical EPCK components, categories, variables, and examples**.

**Theoretical EPCK component**	**Category**	**Variable**	**Level of EPCK**	**Example**
Allocated learning time	No content	1. No content: Off-task/no speech/unintelligible	Non-EPCK	Please be quiet.
Teaching style	Level of Openness^a^	2. Think-time 3. Evoking conceptual understanding	High EPCK	[Silence after question or encouragement] Why do you think it is dark?
		4. Evoking declarative knowledge 5. Evoking procedures 6. Information, instruction or confirmation	Non-EPCK	What planet is this? Where did you attach the tube? This is a salt crystal.
Instructor's reaction to pupil's contribution	Type of reaction	7. React to spontaneous contribution 8. React to contribution (non-spontaneous)	High EPCK	Indeed, this is a mineral. That is indeed the answer.
		9. No reaction to spontaneity 10. Instructor's initiation	Non-EPCK	[No reaction of the instructor after spontaneity] [Instructor starts a new topic/interaction]
Instructor's reaction to pupil's conception	Appearance of reaction	11. No reaction to pupil's (non)conception	Non-EPCK	[No reaction of the instructor after pupil's utterance]
	Judgments	12. Neutral judgment	High EPCK	Okay.
		13. Positive judgment 14. Negative judgment	Non-EPCK	Indeed. No, that is not entirely true.
	Type of feedback	15. Feedback by means of follow-up question	High EPCK	And what will happen then?
		16. Feedback by means of explaining 17. No feedback after judgment	Non-EPCK	Well, what you see here…[after an utterance of a pupil] [No follow-up reaction after the judgment of the instructor]
Pupil's complex thinking	Level of complexity^b^	18. Conceptual understanding	High EPCK	It becomes dark, because the Sun does not shine on that part.
		19. Declarative knowledge 20. Non-complex 21. Procedures	Non-EPCK	That is Saturn. Yes. You have to attach the tube there.
Pupil's contribution	Appearance contribution	22. Spontaneous reaction 23. React to question instructor	High EPCK	I have seen these mills in the harbor! Because it is cold.
Pupil's conception	Type of conception	24. Incorrect conception 25. Fragmented conception 26. Correct conception	High EPCK	The black spots on the Moon are water. What are these spots on the Moon? The spots on the Moon are craters with lava.

*An elaborated Dutch version of the coding system can be requested from the author*.

a *Based on Openness Scale (Meindertsma et al., 2012)*.

b *Based on Skill Theory Scale (Meindertsma et al., 2012; Van der Steen et al., 2012)*.

To determine the reliability of the current study we used the underlying variables of each theoretical EPCK component (see Table 2), and we left out any missing data of the variables. The Kappa's of the theoretical EPCK components were: Allocated learning time 0.85; Teaching style 0.81; Instructor's reaction to pupil's contribution 0.68; Instructor's reaction to pupil's conception 1.00; Pupil's contribution 0.79; Pupil's complex thinking 0.71; Pupil's conception 0.93. These Kappa's revealed that the coding was sufficiently reliable (Viera and Garrett, 2005). Finally, all data used in this study have been checked by the second rater for consistency.

### Data-analyses

To answer the research questions, we used two consecutive analyses for each question. In the case of the first research question (*Can multiple teaching and learning variables be reduced to a limited number of underlying teaching and learning components dimensions, and if so, are these underlying dimensions components idiosyncratic or general across different instructors and out-of-school activities?*), we examined the possibility of reducing the 26 observational variables measured at the micro level (cf. Figure 2) to a limited number of components by using a principal component analysis (PCA) on the time-serial data. Firstly, the data was smoothed with a Loess smoothing technique (Simonoff, 1996) with a window of 10%, which provided us continuous data. We imported the smoothed data from Excel into the software Tanagra^1^ to carry out a PCA for every individual case. We used a factor rotation to improve the interpretability (Beavers et al., 2013). To decide on the amount of factors (components), we stated that the explained variance should at least add up to 50%, and that each factor should have an eigenvalue > 1.00. We chose 50% as a rule of thumb as human behavior will ultimately show a considerable amount of unpredictable variance. Although, the cutoff point is always arbitrary, we considered a factor that explains 50% or more of the variance sufficiently important. We expected that underlying variables interact and that three uncorrelated rotated factors should be sufficient to divide the data into interpretable components. In this way, we would still retain a satisfying amount of explained variance to find discriminating components describing the intertwined teaching and learning process on the micro level (cf. Spicer, 2005; Beavers et al., 2013). Whenever the factors jointly accounted for less than 50% of the variance, a fourth factor with an eigenvalue of at least 1 was added.

The second analysis for answering the first question is a Kohonen's self-organizing maps (SOM) technique (Kohonen, 1982) in the datamining program Tanagra. Kohonen clustering is a technique to lower the dimensions of high-dimensional data. The most interesting aspect of SOM is that the analysis is based on the self-organization of patterns. The technique reduces and categorizes high dimensional data, which makes it possible to detect states into which the teaching and learning processes self-organize. We used this analysis to find similarities between idiosyncratic case components, so that we could find general descriptions of components that can be used across cases. For the clustering technique, we used the correlations of the 26 variables with the case components of all cases (resulted from the first analysis) to find general conceptualizations of the case components. We used the correlations of the variables and the components to retain the time-related information of the idiosyncratic components of individual cases. In order to find out to what extent and how the idiosyncratic components show similarities, we applied a cluster analysis to the correlations between the observed variables and the idiosyncratic components. This means that the case components in one cluster are grouped because they show similar time-related correlations of variables. To facilitate interpretation, we used three clusters to find three general descriptions expressing a level of EPCK. By means of Tanagra, the contribution of particular variables to a cluster of variables can be numerically specified in the form of an indicator called the test value (TV). The TV shows the weight of each variable in the cluster, where higher absolute values indicate a higher weight. The higher the absolute value of the TV of a particular variable, the greater the variable contributes to the classification of observed cases in the cluster in question. The TV is the result of a test of a comparison of means (the mean across the whole dataset compared to the mean in each cluster or state). The Cohen's *d* effect size (ES) shows how much the TV of the variable in the cluster differs from the values of the variable in the other clusters. The ES is determined by the difference score of the average correlation of the variable in the cluster and the average correlation of the variable in the other clusters, divided by the pooled standard deviation of the variables in all clusters. The variables with the largest TV's, the highest ES's (Cohen's *d* > 0.85), and with *p* < 0.05 are considered indicators for the conceptualization of the components in the cluster. To estimate the probability that the ES was caused by chance alone, a Monte Carlo analysis (Good, 2001) was used. This non-parametric permutation test uses randomization (we used 2,000 permutations) and is used in combination with Excel and PopTools (Hood, 2010). By randomly assigning the values of variables to the components in the clusters, simulated ES were calculated. The outcome corresponded with our null hypothesis that there would be no statistically significant differences between the observed ES and the simulated ES. A statistical significance was expressed in terms of *p*-values. 95% confidence intervals of effect sizes observed under the null hypothesis and 95% confidence intervals of the observed effect size ware calculated.

To find out how successive moments in an actual out-of-school activity cluster into qualitatively distinct, successive teaching and learning states, which are potential attractor states, and to find out how comparable the self-organizing processes of these states across cases are (question 2), we first carried out the Kohonen's self-organizing maps (SOM) technique to the data of the individual cases by using the time series of the components. The analysis revealed the emergence of structure in time-serial data (De Ruiter et al., 2017)—in this case the emergence of EPCK states on the meso level (cf. Figure 2). We used three clusters to find discriminating EPCK states of considerable size, as we considered this the best possible compromise between generality and detail given the duration of the observation. The analysis reveals what percentage of time the state was active during the observation. The TV indicates which components are dominant in the state. For each EPCK state, the component with the highest positive or negative TV is considered to be the component that is most likely to activate the self-organization of that specific state. The ES shows how the TV of the component in a particular state differs from the values of the factor in the other states. Whenever the system changes from one state to another, i.e., during a transition period, the case components show a significant increase or decrease. These transitional values of the components were not included in the characterization of the states or in the calculation of the difference between those states in terms of ES—we left out 15% of the values at the starting point of the state occurrence and another15% at the end of the state occurrence. We defined a component as dominant whenever the ES was very large (*d* > 0.85/*d* < −0.85) and had a *p* < 0.001. To calculate the *p*-value and the 95% confidence intervals of effect sizes, we used the Monte Carlo analysis. First, we shuffled rows of each time point over all clusters and then compared the ES of the simulated data with the observed data under the null hypothesis that there was no difference. Second, we resampled (with replacement) the rows of each time point within the clusters, to establish the 95% confidence intervals of the observed effect sizes.

Next, we inspected the EPCK levels of the states: high, low or no EPCK. To facilitate interpretation of the clusters in terms of EPCK, we have only concentrated on the positive values of the components. From an EPCK theory perspective, *High EPCK* is preferable above *Low* or *Non-EPCK*. This means that only if the component High EPCK is strongly positively associated with the state or cluster (i.e., if that component has a high positive TV), the teaching and learning that occurs in the state or cluster shows High EPCK. Negative domination of this component (i.e., high negative TV's) corresponds with forms of learning in which EPCK is typically absent: Non-EPCK. All other variants were considered to be Low EPCK. Because we expected that high-level EPCK may be accompanied by a variety of additional properties, we decided not to distinguish between mixed high-low level states and only high-level states, and to specify the state by its highest outcome (EPCK theoretically). Whenever there is a balance in the loads, the state cannot be characterized as either High EPCK, Non-EPCK, or Low EPCK.

In order to answer the third question *(What are characteristics of the attractor states, if any are found, and can the data be related to the properties of different out-of-school activities?)*, we first inspected the states for theoretical properties for attractor states: recurrent pattern of each state, state durations and the variability of the dominating components in the state. Based on theoretical argumentation we stated that for a state to be interpreted as an attractor state, it needs to reoccur three or four times within the 600 coded s to show both recurrence and substantial duration time per recurrence. Another criterion for treating a particular state as an attractor state was based on the variability of the components in a state, by calculating the coefficient of variation (CV) of the components in a particular state. If the variability of the dominant component was smaller than the average CV of all components in that state, we considered the component to be sufficiently stable, thus indicating that the state is an attractor state.

Lastly, to compare the states on the basis of additional case properties (see Table 1), we inspected the conceptualization of the attractor states (High EPCK, Low EPCK or no EPCK). We then compared the cases in terms of the type of activity, whether the pupils were prepared or not, and whether the instructor had followed the short training in EPCK or not.

## Results

### Teaching and learning components in out-of-school activities (micro)

The first question was: *Can multiple teaching and learning variables be reduced to a limited number of underlying teaching and learning components, and if so, are these underlying components idiosyncratic or general across different instructors and out-of-school activities?* The PCA of the individual cases reduced the data to three components (in six cases) or to four components (in three cases). The total variance explained by components in each case was between 55 and 68% (see Table S1). The components were characterized on the basis of interacting variables on the micro level. Although, the components were idiosyncratic, typical components also showed typical similarities. The Kohonen clustering technique revealed that the particular case components could be described in a similar vein, resulting in three general descriptions. Table 3 shows the characterization of these general descriptions and the components that fit the descriptions. The first row shows which components were included in each cluster. The first column of each cluster shows the variables that make a major contribution to the cluster. The second and third columns of each cluster show the TV's and ES's of each variable in each cluster. The variables with high, positive TV's and with large ES's (Cohen's *d* > 0.85) reveal a general description that characterizes all components in the cluster.

**Table 3:** **Empirical findings with regard to clustering components by variables of the case factors**.

**Cluster 1**						**Cluster 2**						**Cluster 3**					
**(Case1_C1, Case2_C1, Case3_C2, Case4_C1, Case5_C1, Case7_C3, Case8_C1)**						**(Case1_C3, Case1_C4, Case2_C3, Case3_C1, Case4_C3, Case5_C3, Case6_C1, Case7_C4, Case8_C3, Case9_C1, Case9_C3)**						**(Case1_C2, Case2_C2, Case3_C3, Case4_C2, Case5_C2, Case6_C2, Case6_C3, Case7_C1, Case7_C2, Case8_C3, Case8_C4, Case9_C2)**					
**Characterizing variables**	**TV**	***d***	***p***	**95% CI**^b^		**Characterizing variables**	**TV**	***d***	***p***	**95% CI**^b^		**Characterizing variables**	**TV**	***d***	***p***	**95% CI**^b^	
				**Upper**	**Lower**					**Upper**	**Lower**					**Upper**	**Lower**
Feedback by means of follow-up question^a^	3.67	2.19	<0.001	−0.84	0.91	Declarative knowledge	2.33	0.98	0.008	−0.76	0.79	Spontaneous reaction^a^	4.03	2.31	<0.001	−0.78	76
				1.16	4.46					0.27	2.02					1.46	3.91
Evoking conceptual understanding^a^	3.49	2.19	0.001	0.01	0.92	React to non-spontaneous contribution^a^	2.21	0.92	0.018	−0.74	0.84	No reaction to spontaneity	3.36	1.62	<0.001	−0.76	0.76
				0.96	4.45					0.15	2.06					0.95	1.56
Conceptual understanding^a^	3.00	1.57	0.001	−0.79	0.94							No reaction to pupil's (non)concept	3.07	1.40	0.001	−0.77	0.77
				0.61	3.58											0.76	2.38
Think-time^a^	2.92	1.51	0.001	−0.83	0.96							Non-complex thinking	2.78	1.22	0.001	−0.78	0.78
				0.37	4.23											0.53	2.40
Incorrect concept^a^	2.41	1.17	0.007	−0.87	0.87							React to spontaneous contribution^a^	2.61	1.12	0.002	−0.75	0.74
				0.43	2.33											0.34	2.30

*Only variables with a large effect size (d > 0.85; p < 0.05) were included in the table. C1, C2, C3, and C4 refer to the first, second, third, or fourth component*.

a *High-EPCK variable*.

b *95% CI resp. 95% CI of effect sizes observed under the null hypothesis and 95% CI of the observed effect size*.

Based on Table 3, the components in Cluster 1 as the combination of *Open teaching focused on eliciting conceptual understanding and pupils' conceptual (mis)understanding* (General Description 1). This general description encompasses the following properties: the instructor's evoked conceptual understanding through asking questions and expressed encouragement of pupils to think out loud, the think-time provided by the instructor after a thought-provoking question, feedback given by the instructor in the form of follow-up questions; and pupils' expressed conceptual understanding and misconceptions (e.g., a wrong explanation). See Table 2 for examples of utterances that are related to the variables. The components in Cluster 2 can be described as *Declarative knowledge and reaction to non-spontaneity* (General Description 2). This general description encompasses pupils' declarative knowledge (e.g., a definition) and the instructor's reaction to contributions of pupils that were not spontaneous but elicited by questions of the instructor. The components in Cluster 3 can be described as *Spontaneity and non-complex thinking* (General Description 3). This general description encompasses pupils' spontaneous contributions to the conversation, which were typically non-complex (e.g., “yes”) and was either acknowledged or not (i.e., the instructor did or did not respond). It is salient that Cluster 1 showed high TV's on five High-EPCK variables, whereas the other clusters only contained one or two High-EPCK variables. Consequently, we interpret components with General Description 1 as representing High EPCK, and components with General Description 2 and 3 as representing Low EPCK.

In conclusion, the answer to question 1 is that multiple teaching and learning variables can be reduced to distinct teaching and learning components. Although, these case components were idiosyncratic, they also showed commonalities across cases (as we expected). The clustering technique revealed that all case components can be characterized as one of the three general descriptions, and that these general descriptions express either High or Low EPCK.

### Forming of teaching and learning states in out-of-school activities (meso)

The Kohonen cluster analysis was used to answer the second question *How do successive moments in an actual out-of-school activity cluster into qualitatively distinct successive teaching and learning states, and how comparable are the self-organizing processes of these states across cases?* It revealed that there were three EPCK states per case on the meso level, which can be interpreted as self-organizing patterns of case components on the micro level. To illustrate how the components self-organize into states, we used Case 5 and Case 8 as examples. Both cases concerned an activity in the Mobile Classroom (Salt Express). The instructor is the same in both cases, but the pupils are different. Table 4 shows the results of Case 5.

**Table 4:** **Components and EPCK states of Case 5**.

**Characterizing components**	**EPCK State 1 (26.2%)**					**EPCK State 2 (36.2%)**					**EPCK State 3 (37.7%)**				
	**TV**	***d***	***p***	**95% CI**^a^		**TV**	***d***	***p***	**95% CI**^a^		**TV**	***d***	***p***	**95% CI**^a^	
				**Lower**	**Upper**				**Lower**	**Upper**				**Lower**	**Upper**
Case5_C1_GD1	5.08	0.33	0.001	−0.22	0.21	7.04	0.81	<0.001	−0.20	0.19	−11.59	−1.14	<0.001	−0.19	0.21
				0.11	0.55				0.59	1.04				−1.32	−0.97
Case5_C2_GD3	18.92	3.01	<0.001	−0.21	0.22	−9.13	−0.84	<0.001	−0.20	0.21	−8.11	−0.74	<0.001	−0.21	0.21
				2.69	3.38				−0.94	−0.74				−0.86	−0.61
Case5_C3_GD2	8.70	1.22	<0.001	−0.21	0.22	3.16	0.21	0.05	−0.19	0.21	−11.03	−1.26	<0.001	−0.19	0.19
				0.99	1.47				0.04	0.41				−1.41	−1.14

*C1, C2, and C3 refer to the first, second, or third component. GD1, GD2, and GD3 refer to the first, second, or third general description. Components with a large effect size (d > 0.85/d < −0.85; p < 0.001) are dominant*.

a *95% CI resp. 95% CI of effect sizes observed under the null hypothesis and 95% CI of the observed effect size*.

It is clear that EPCK State 1 was primarily characterized by Component 2 with General Description 3 Spontaneity and non-complex thinking, which indicates Low EPCK. This particular interaction state occurred in 26.2% of the total interaction time. As for EPCK State 2, which had a total duration of 36.2% of the observation, no particular general component was dominant. Consequently, this state could not be characterized by one of the general descriptions. EPCK State 3 was negatively dominated by Component 1 with General Description1 Component 3 with General Description 2, indicating that this state was typically the opposite of “Open teaching focused on eliciting conceptual understanding and pupils' conceptual (mis)understanding” and “Declarative knowledge and reaction to non-spontaneity.” This indicates that EPCK State 3 is a Non-EPCK state, because no “Open teaching focused on eliciting conceptual understanding and pupils' conceptual (mis)understanding” was found. This state occupied a total of 37.7% of the observed time.

**Figure 3:**
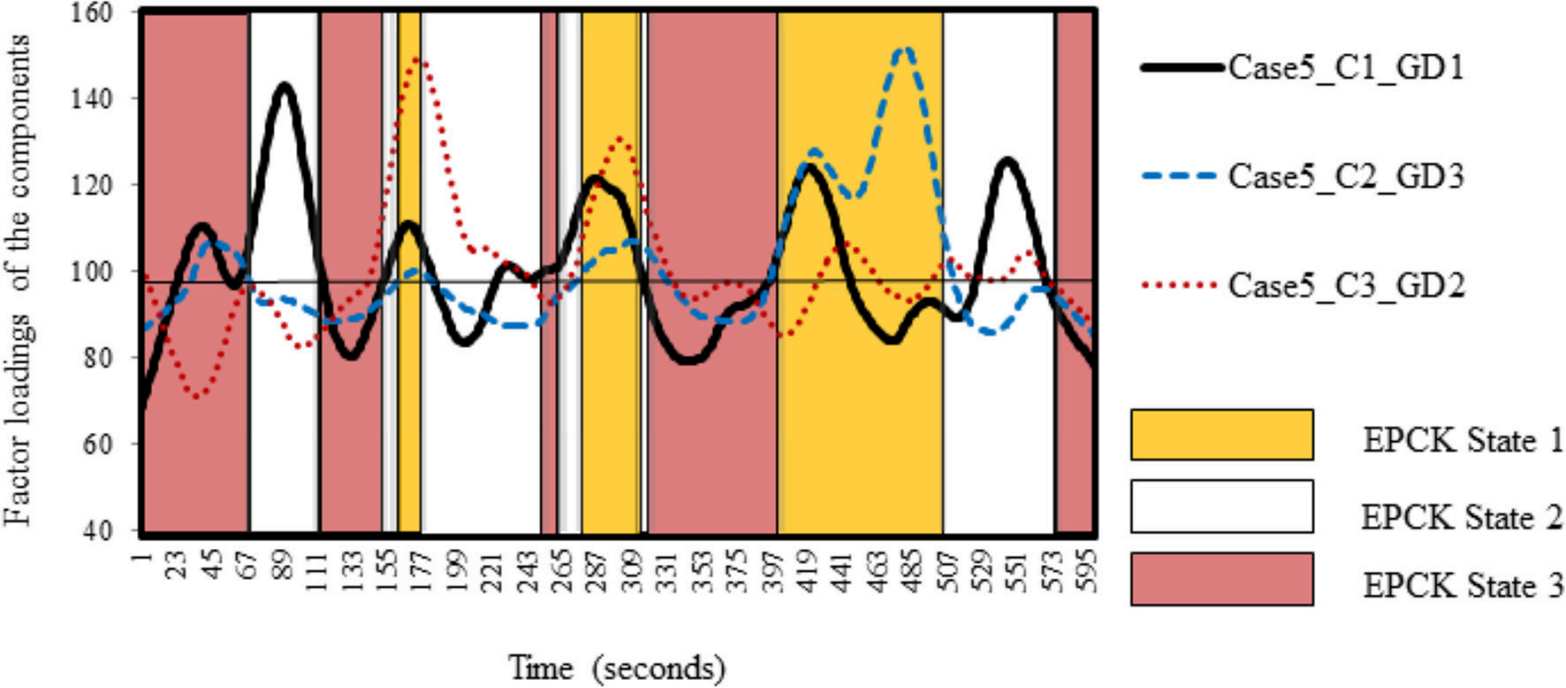
**Successive EPCK States in Case 5**. Self-organizing patterns of the components in Case 5 distributed over time, revealing distinct successive EPCK states. Average factor loading of the components is 100. C1, C2, and C3 refer to the first, second, or third component. GD1, GD2, and GD3 refer to the first, second, or third general description.

Figure 3 shows a temporal distribution of these components and the states of Case 5. The timescale is on the x-axis, while the values of the components are on the y-axis. The lines represent the distributed components over time.

This figure shows the successive moments of the actual out-of-school activity and its distinct successive teaching and learning states. These states emerge as a result of the interaction on the micro level (patterns of the case components). These self-organizing patterns are different for each state. To illustrate this, EPCK State 1 (yellow shaded) was characterized as “Spontaneity and non-complex thinking,” although this was merely due to the peak in Component 2 with General Description 3 in the last occurrence of the state. The emergence of EPCK State 3 (red shaded) on the other hand, which was characterized as a Non-EPCK state, was the result of the drops of General Component 1 during the second, fourth and last occurrence of the state, and the drops of Component 1 with General Description 1 during almost each occurrence of the state. Finally, EPCK State 2 (non-shaded) could not be characterized by a described by means of a general description due to the absence of dominant components, although Component 1 with General Description 1 (Open teaching focused on eliciting conceptual understanding and pupils' conceptual (mis)understanding) peaked in the first and last occurrence of EPCK State 2. Apparently, this peak was not enough to cause self-organization.

Table 5 shows the results of the cluster analysis of Case 8.

**Table 5:** **Components and EPCK states of Case 8**.

**Characterizing components**	**EPCK State 1 (26.2%)**					**EPCK State 2 (36.2%)**					**EPCK State 3 (37.7%)**				
	**TV**	***d***	***P***	**95% CI**^a^		**TV**	***d***	***p***	**95% CI**^a^		**TV**	***d***	***p***	**95% CI**^a^	
				**Lower**	**Upper**				**Lower**	**Upper**				**Lower**	**Upper**
Case8_C1_GD 1	−14.54	−1.61	<0.001	−0.23	0.23	−3.98	−0.42	<0.001	−0.19	0.19	17.91	2.21	<0.001	−0.20	0.21
				−0.182	−1.43				−0.60	−0.24				2.02	2.42
Case8_C2_GD 3	11.22	1.85	<0.001	−0.24	0.24	−12.44	−1.27	<0.001	−0.19	0.18	2.26	0.06	0.56	−0.21	0.22
				1.61	2.11				−1.47	−1.08				−0.11	0.24
Case8_C3_GD 2	−4.53	−0.23	0.005	−0.23	0.23	−0.14	−0.21	0.018	−0.20	0.19	4.42	0.43	<0.001	−0.21	0.21
				−0.44	−0.01				−0.39	−0.03				0.20	0.66
Case8_C4_GD 3	5.33	0.72	<0.001	−0.23	0.23	−11.96	−0.96	<0.001	−0.19	0.18	7.66	0.45	<0.001	−0.20	0.21
				0.45	1.01				−1.11	−0.83				0.24	0.69

*C1, C2, C3, and C4 refer to the first, second, third, or fourth component. GD1, GD2, and GD3 refer to the first, second, or third general description. Components with a large effect size (d > 0.85/d < −0.85; p < 0.001) are dominant*.

a *95% CI resp. 95% CI of effect sizes observed under the null hypothesis and 95% CI of the observed effect size*.

The table shows that EPCK State 1 of Case 8 was positively dominated by Component 2 with General Description 3 and negatively dominated by Component 1 with General Description 1. This indicates that EPCK State 1 is a Non-EPCK state. This particular interaction state occurred in 26.0% of the total interaction time. In EPCK State 2, Component 2 and Component 4 both with General Description 3 were negatively dominant, which indicated Low EPCK that could only by characterized as typically not being “Spontaneity and non-complex thinking.” This state occupied a total of 42.2% of the observed time. EPCK State 3 was positively dominated by Component 1 with General Description 1, indicating High EPCK. 31.8% of the time this interaction was in this state.

**Figure 4:**
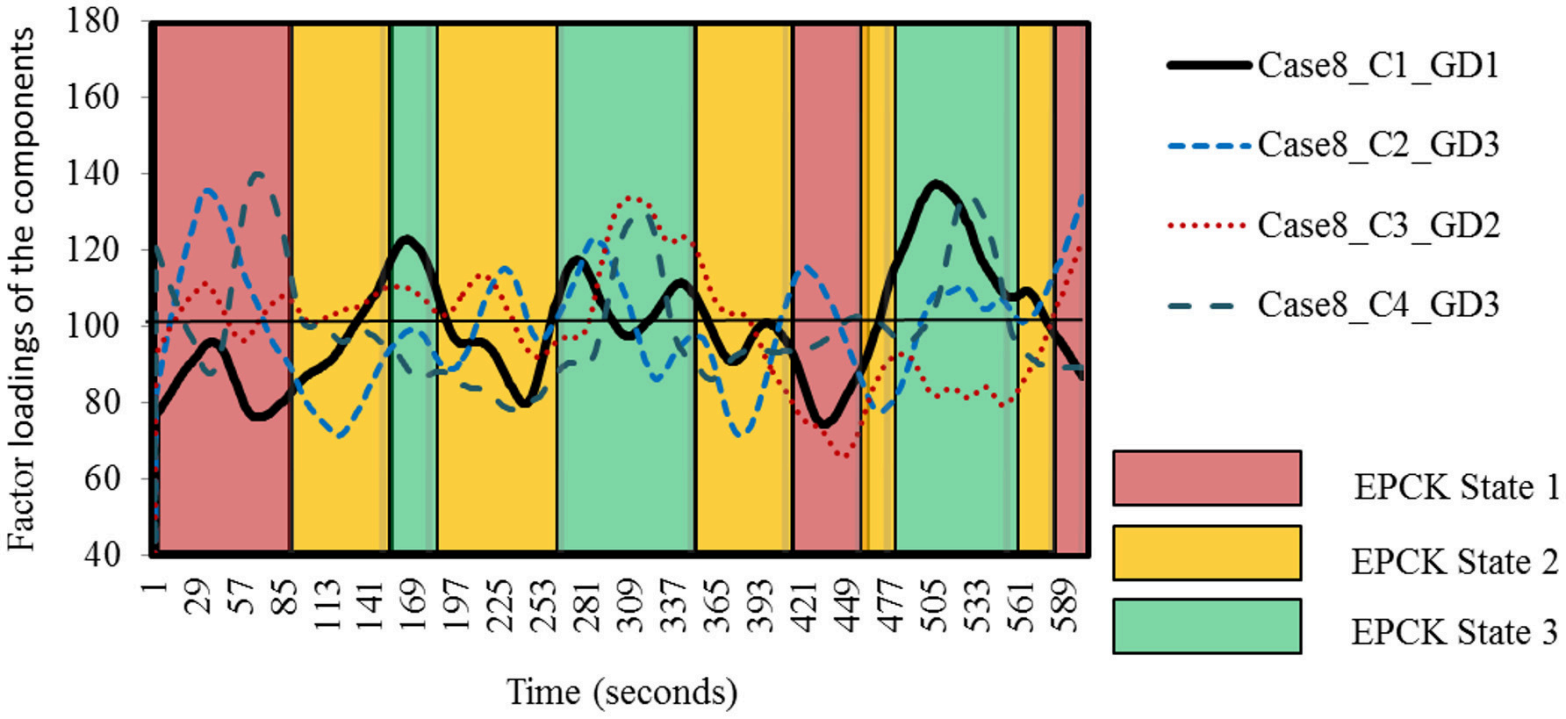
**Successive EPCK states in Case 8**. Self-organizing patterns of the components in Case 8 distributed over time, revealing distinct successive EPCK states. Average factor loading of the components is 100. C1, C2, C3, and C4 refer to the first, second, third, or fourth component. GD1, GD2, and GD3 refer to the first, second, or third general description.

Similar to Figures 3, 4 shows the self-organizing patterns of the components, revealing its successive states. These patterns show that there is either a peak/drop of the component in several state occurrences but not in all (in EPCK State 2) or there is a peak/drop in each state occurrence (in EPCK State 1 and EPCK State 3).

Applying the Kohonen clustering technique to all cases, we found that five out of 27 states were positively dominated by components with General Description 1, indicating High EPCK. In six states components with General Description 1 were negatively dominant, indicating Non-EPCK. In 10 states components with General Description 2 and/or General Description 3 were positively dominated, indicating Low EPCK. In four states General 2 or 3 were negatively dominant, also indicating Low EPCK. In 2 states no dominating components were found, due to the variable pattern of these components.

As we expected, components self-organize into successive states of High EPCK, Low EPCK or Non-EPCK, at least in most of the cases. These states either show a large peak/drop in the dominating components during each occurrence, or show a large peak in several (but not all) occurrences. Thus, peaks/drops in components do not by definition cause self-organization. Idiosyncrasy was found in two states, due to the variable pattern of the components on the micro level. Additionally, although four Low-EPCK states show similarities in that they do not represent “Declarative knowledge and reaction to non-spontaneity” and/or “Spontaneity and non-complex thinking,” such states are also characterized as typically idiosyncratic in what they do present.

### EPCK attractor states (macro)

Our third question was *What are the characteristics of the attractor states, if any are found, and can the data be related to the properties of different out-of-school activities?* Table 6 shows (for each case) which states revealed a sufficiently recurrent pattern on the meso level of three or four recurrences, guaranteeing a substantial duration. It also shows which states contained at least one dominant component with low variability (Coefficient of variation; CV) on the meso level. This low variability is a requirement for the assignment of an attractor quality to the states on the macro level. The levels of EPCK (High, Low or Non-EPCK) are listed in the bottom row of Table 6.

**Table 6:** **Characteristics of (Potential) attractor states and case properties**.

	**Case 1^e^^,^^f^**	**Case 2^e^**	**Case 3**	**Case 4^e^^,^^f^**	**Case 5**	**Case 6^f^**	**Case 7**	**Case 8^e^^,^^f^**	**Case 9**
**STATE 1**									
Occurrence	3	2^a^	2^a^	3	3	4	3	3	4
Duration	233	71	73	119	157	170	283	156	213
GD first dominant component	1^b^	1	1	1^b^	3	2^neg^c^^	3^neg^c^^	1^neg^d^^	2^c^
GD second dominant component		3			2	3^neg^	2^neg^	2	
CV first dominant component	0.10	0.20^a^	0.05	0.06	0.14	0.07	0.06	0.09	0.07
		0.23^a^			0.16^a^	0.05	0.09	0.09	
CV second dominant component									
**STATE 2**									
Occurrence	4	3	2^a^	4	6^a^	4	2^a^	5^a^	5^a^
Duration	182	61	91	351	217	265	256	253	332
GD first dominant component	1^neg^d^^	—^a^	2	1^neg^d^^	—^a^	3^c^	2	3^neg^	2^neg^
	2			3^neg^				3^neg^	3^neg^
GD second dominant component									
CV first dominant component	0.07	—	0.12	0.06	—	0.12	0.14	0.13	0.05
	0.06			0.09				0.07	0.12
CV second dominant component									
**STATE 3**									
Occurrence	2^a^	3	3	3	5^a^	3	1^a^	3	1^a^
Duration	185	468	436	130	226	165	61	191	55
GD first dominant component	3	1^neg^d^^	1^neg^d^^	3^c^	1^neg^	2	3	1^b^	2^neg^
GD second dominant component	2^neg^	3^neg^	2^neg^		2^neg^	3^neg^			3
									2^neg^
CV first dominant component	0.18^a^	0.03	0.01	0.11	0.11	0.10	0.03	0.09	0.01
CV second dominant component	0.21^a^	0.05	0.02		0.09	0.21^a^			0.06
									0.03
**TOTAL**									
CV	0.16	0.15	0.16	0.16	0.16	0.16	0.16	0.14	0.15
Number of attractor states	2	1	1	3	0	2	1	2	1
Level of EPCK attractor states	High; Non	Non	Non	High; Non; Low	-	Low; Low	Low	Non; High	Low

*GD, General Description; CV, Coefficient of variation*;

neg, *negatively dominant*.

a *Indicator of no attractor state*.

b *High-EPCK attractor state*.

c *Low-EPCK attractor state*.

d *Non-EPCK attractor state*.

e *Pupils were prepared*.

f *Instructors were trained*.

Based on Table 6, we can conclude that in 8 out of 9 cases at least one state develops into an attractor state during a single observation (13 attractor states in total). We found five examples of High-EPCK states, although there were only three cases in which this state is revisited often enough and shows sufficient duration and stability to deserve consideration as an attractor state. Of the six Non-EPCK states, five turned out to be attractor states, according to the criteria of frequency, duration, and variability. The five remaining attractor states could be characterized as Low EPCK.

We were interested in comparing the cases on the basis of the type of activity, whether the pupils had been prepared for the activity or not, and whether the instructor had been trained for the application of High EPCK or not. In Table 1 (method section) we displayed an overview of the case properties. After comparing the properties with the results in Table 6, we found that attractor states of High EPCK occurred only in those cases where pupils had been prepared and instructors were trained (Case 1, Case 4, and Case 8). When pupils had not been prepared and the educator was not trained, only attractor states of either Low or Non-EPCK occurred. On the other hand, no connection could be found between the occurrence of particular attractor states (e.g., High EPCK) and the type of out-of-school activity.

As we expected, we were able to find attractor states on the macro level. These attractor states indicate episodes of relatively persistent and temporarily self-sustaining teaching and learning processes of High EPCK, Low EPCK, or Non-EPCK. These EPCK attractors occurred during particular episodes of the activities rather than being present all the time. There are indications that preparing pupils and training instructors matters when it comes to high-quality science teaching and learning. There seems to be no correlation between the occurrence of EPCK attractors and the type of activity. These findings are consistent with our expectations about idiosyncrasy and commonality.

## Conclusion and discussion

This study focused on the self-organization of teaching-learning processes in an attempt to find Expressed Pedagogical Content Knowledge (EPCK) attractor states and their possible connection to particular out-of-school activities. As attractor states are developed during interactions on the micro level, we wanted to know firstly whether multiple teaching and learning variables can be reduced to idiosyncratic teaching and learning components, components that express various levels of EPCK, and secondly whether such idiosyncratic components can be conceptualized in a more general way. By applying a principal component analysis to 26 observed variables, the interaction became interpretable on the micro level in terms of a small number of underlying components. By means of a variable-clustering technique we were able to conceptualize the idiosyncratic components in a general manner, resulting in three general descriptions: Open teaching focused on eliciting conceptual understanding and pupils' conceptual misunderstanding (High EPCK), Declarative knowledge and reaction to non-spontaneity (Low EPCK), and Spontaneity and non-complex thinking (Low EPCK). We measured the data of nine cases on the micro level and used them to find out how teaching and learning components self-organize into states within cases on the meso level, i.e., within particular out-of-school activities spanning a duration of 10 min. The Kohonen clustering technique was applied to individual cases in order to find clustering of components into EPCK states. Each case revealed unique combinations of components, as well as idiosyncrasy of successive EPCK states. We found that dominant components self-organize into states, either by peaking/dropping in several occurrences of a particular state or by peaking/dropping in each occurrence of the state. Similarities were found in the characterization of states, in that the dominant components revealed High ECPK (5), Low EPCK (14), or Non-EPCK (6) on the meso level. Only two states were entirely idiosyncratic. Finally, in order to find attractor states on the macro level, we wanted to know what the characteristics of the attractor states were in relation to the properties of different out-of-school activities. We found that states of substantial duration, with recurrent patterns, and with low variability of dominant components (which we defined as attractor states) could be conceptualized as High EPCK, Low EPCK or Non-EPCK. This attractor states alternated indicating that High EPCK occurs only during particular episodes within an activity, rather than being present all the time. High EPCK attractor states were only found in three cases, namely those in which the pupils had been prepared for the visit and instructors were trained. There seems to be no relation with the type of out-of-school activity and the type or pattern of attractor.

The majority of the findings, especially those with regard to idiosyncrasy of cases and commonality between cases, were consistent with the expectations and in accordance with a number of previous studies on Pedagogical Content Knowledge (e.g., Crowley et al., 2000; Park and Oliver, 2007; Bamberger and Tal, 2008; Park and Suh, 2015). Although, in these studies Pedagogical Content Knowledge (PCK) is perceived as a quality of the teacher, they do share our point of view that high-quality education always takes place in the context of pupils' behavior. Regarding the development of EPCK from micro level to macro level, Seymour and Lehrer (2006) argued that high-quality teaching cannot emerge at once but must evolve over the course of several lessons. A salient finding from our study however is that High EPCK occurs in stable sequences, measured during a single visit. Seymour and Lehrer (2006) defined “orchestrate classroom” as an important aspect of PCK, in which teachers' practices are attuned to pupils' needs. In our study, this attunement is typically present in High-EPCK attractor states that are characterized by “Open teaching focused on eliciting conceptual understanding and pupils' conceptual (mis)understanding.” These moments of uplifting High EPCK are comparable with “learnable moments” and “teachable moments” (DeWitt, 2012; Haug, 2014) in which pupils are helped toward conceptual understanding by the educator and motivated to learn particular science concepts. In this study high EPCK corresponds—on average—with conceptual understanding in the pupils, and thus conceptual understanding occurs considerably less frequently in the states that are not high EPCK. We may conclude that high EPCK corresponds with a type of high-level reasoning of which teachers wish to increase by using high quality teaching process. A sustainable increase in such high-level reasoning would be a form of learning itself.

Virtually all previous studies that were focused on the idiosyncrasy and developmental nature of PCK did not explicitly use the concept EPCK. By making a distinction between PCK and EPCK and by focusing on the latter, our study explicitly states that PCK is something that must be conceptualized in terms of real-time events. Studying the dynamic pattern in which EPCK is constructed on the meso and macro levels demonstrated that each case shows its own pattern of states, i.e., case-specific dynamics of the interaction between instructor and pupils on the micro level. However, it is also important to stress that, in spite of the idiosyncratic nature of EPCK, similarities were found between cases in terms of correlations between EPCK variables and a small number of components.

Various studies have acknowledged that PCK is constructed during (inter)action in the classroom (Seymour and Lehrer, 2006; Park et al., 2011). These studies make use of a qualitative approach to investigate the emergence of PCK. The added value of a quantitative method is that the relation between the micro, meso and macro levels can also be used to find evidence for patterns in long-term development (interconnected timescales). Long-term development of PCK has been investigated by using microgenetic approaches. Although, PCK studies use reflection methods (vignettes) to find changing patterns in knowledge and beliefs of teachers (e.g., Luft and Zhang, 2014). These methods do not reflect the actual behavior in the classroom. In our view, PCK should primarily be measured in action, as that is what actually influences pupils. Moreover, pupils form the dynamically intertwined context in which PCK is dynamically constructed.

This study has three limitations. The first limitation is that the total explained variance of the components in each case was between 55 and 68%, which means that there is still a large residue of unexplained variance. Still, at least more than half of the variance could be explained by just three or four components, of which one was in accordance with the theoretical construct of High EPCK. Although, using more components in the data reduction would enlarge the total variance, it would also make it harder to interpret the data, resulting in a loss of theoretical focus. It is clear that the intertwined teaching and learning process is too complex to capture a large amount of variance in a limited number of components. The second limitation is that the long-term development of EPCK could not be investigated, as an out-of-school science activity is usually a one-time event (Popovich and Zint, 2012). The scant amount of existing studies on long-term development and out-of-school learning are usually about memory retrieval of the actual visit (e.g., Knapp, 2007) and do not investigate long-term teaching and learning processes. Using the dynamic systems approach, development of EPCK could be explored in out-of-school science activities by making repeated measurements of multiple visits. For instance, we could have compared Case 5 and Case 8 with regard to the development of EPCK, as these cases involved the same activity and the same instructor. It is clear for instance that in Case 5—when the instructor was not yet trained—High EPCK was only found at the micro level, whereas a year later—after the instructor had received training—EPCK had developed into a High EPCK attractor state on the macro level. We have chosen not to include these findings in the results, however, as the pupils involved in these cases were from different schools. As we argue that the development of EPCK is a process constituted by the interaction between instructor and pupils, these cases were not suitable enough in our view to measure long-term development of EPCK. A better way to investigate long-term EPCK development would be to use observations of a particular instructor with a particular group of pupils who are following a long-term project consisting of multiple visits. As we did not have these kinds of projects at our disposal, we could not investigate long-term development in this study. However, it would be interesting to do so in future research. The third limitation is that in this study we used general as opposed to topic-specific variables to indicate EPCK. However, it is mentioned in existing literature that PCK is also considered to be topic- or subject-specific (e.g., Magnusson et al., 1999; Rohaan et al., 2011). Coding topic-specific variables to conceptualize topic-specific EPCK might reveal additional components, though in general the same methodology can be used to reduce the components at the micro level. In this study, we used a general approach to compare cases of a different nature. It is likely that, because of this non-topic-specific approach, the general components found in this study would also be found in other out-of-school activities, showing just slightly deviating conceptualizations.

The practical implication of this study is that the identification of attractor states can be used to improve the teaching and learning process. Different features and levels of EPCK can be detected in educational practices. As the attractor states correspond with the time-serial data on video, these results can be used in video feedback coaching (e.g., VFC-T of Wetzels, 2015) to confront educators with the data concerning their application of high-quality teaching and learning. According to (Leach and Conto, 1999), feedback on the quality of past teaching performances is effective for the improvement of teaching and learning. The results of our analysis identify at what moment a typical form of EPCK was expressed. When interactions of High-EPCK can be detected and extended, potential attractors on the meso level (states) can be intensified. Low-EPCK attractors that show elements of high-level EPCK on the micro level might even develop toward a higher level. Moreover, a repeated combination of undesired behaviors, i.e., Non-EPCK elements, is a sign of “falling” into an attractor. We found such an attractor in many cases in this study. Educators should be aware of such Non-EPCK attracting behaviors, e.g., providing too much information and initiating new topics too often, rather than asking questions and inviting pupils to contribute to the science lesson. Furthermore, this study shows that spontaneity of pupils is an indication of Low-EPCK, due to its occurrence in combination with Non-EPCK variables in real time (e.g., non-complex thinking). However, in teaching practice, contributions of pupils and the spontaneous asking of questions should be encouraged, in order to stimulate pupils' cognitive autonomy (Stefanou et al., 2004) and motivate pupils to learn about science (Berlyne, 1960; Csikszentmihalyi and Csikszentmihalyi, 1988; Osborne et al., 2003). It is a challenge for teachers to elicit higher-order thinking when pupils show spontaneous contributions rather than staying on the lower-order thinking level or ignoring the spontaneous contribution, which was what we observed in several cases in this study. However, when educators do succeed in scaffolding pupils' spontaneous intuitive knowledge or thoughts, they might evoke pupils' revelation (aha-experience) and give them the opportunity to reflect on those insights (Iran-Nejad and Stewart, 2010) in order to deepen conceptual understanding.

Although, this study consisted of a limited amount of cases, we obtained insight into how EPCK is constructed during micro interaction in out-of-school activities and how it self-organizes into (potential) attractor states. Our study reveals how interaction between pupils and instructors changes over the course of one visit, and also how teaching and learning patterns differ across cases on the basis of case properties such as preparing the pupils in advance and training the instructor to give high-quality teaching in interaction with pupils.

## Author contributions

CG: Conceptualizing ideas, conducting the investigation process, design EPCK coding system, coding of videos, developing and designing methodology, interpretation of analyses, design figures and tables, draft of manuscript. HS: Conceptualized ideas, editing of manuscript, interpreting of analyses, general advice on research procedure and writing. JD: Conceptualized ideas, interpreting of analyses, general advice on research procedure and writing, editing of manuscript. PV: Conceptualizing ideas, developing and designing methodology, interpretation of analyses, general advice on research procedure and writing, draft and editing of manuscript.

### Conflict of interest statement

The authors declare that the research was conducted in the absence of any commercial or financial relationships that could be construed as a potential conflict of interest.

## Footnotes

^1^The PCA and the SOM analysis were done in the data mining program Tanagra 1.4.50 (Rakotomalala, 2005). For more information regarding this analysis technique, see the tutorial “Self-organizing map (SOM)” provided by Tanagra (http://data-mining-tutorials.blogspot.nl/2009/07/self-organizing-map-som.html).
