# Supplementary material for: Attractor States in Teaching and Learning Processes: A Study of Out-of-School Science Education
Source: Front Psychol. 2017 Mar 3;8:299. doi: 10.3389/fpsyg.2017.00299 (PMC5334571; doi:10.3389/fpsyg.2017.00299)
Supplement: Supplementary file 1 [file DataSheet1.docx]

# Supplemental material

**Table S1. Principal Component Analysis of all Individual Cases**

| Total explained variance | Component 1  variables corr. >.50 | Component 2  variables corr. >.50 | Component 3  variables corr. >.50 | Component 4  variables corr. >.50 |
| --- | --- | --- | --- | --- |
| Case 1  58% | Feedback by means of follow-up question; Think-time; Evoking conceptual understanding; React to question teacher; Conceptual understanding; Fragmented concept | Incorrect concept; Procedures; Declarative knowledge; Neutral judgment; Spontaneous reactions | Evoking declarative knowledge; No feedback after judgment | React to question teacher; Conceptual understanding; Correct concept; React to non-spontaneous contribution |
| Case 2  63% | Conceptual understanding; Incorrect concept; Fragmented concept; Positive judgment; Think-time; No feedback after judgment; Evoking conceptual understanding; Evoking Procedures; React to non-spontaneous contribution | Spontaneous reactions; Non-complex thinking; Neutral judgment; No reaction to pupils (non)concepts; Evoking conceptual understanding; No feedback after judgment | Information, instruction or confirmation; React to spontaneous contribution; Procedures |  |
| Case 3  68% | React to question teacher; Declarative knowledge; Correct concept; Fragmented concept; Incorrect concept; Off-task/ no speech/ unintelligible; No feedback after judgment ; Incorrect concept | Evoking conceptual understanding  Feedback by means of follow-up question; Positive judgment;  Negative judgment; Conceptual understanding; No feedback after judgment; Think-time | React to non-spontaneous contribution; Feedback by means of explaining; Neutral judgment T); Non-complex thinking |  |
| Case 4  55% | Evoking conceptual understanding; No feedback after judgment ; Positive judgment; Neutral judgment; Conceptual understanding;  Feedback by means of follow-up question; Correct concept; Evoking declarative knowledge | No reaction to pupils (non)concepts  No reaction to spontaneity  Non-complex thinking  Spontaneous reactions | React to non-spontaneous contribution; Declarative knowledge; Positive judgment;  Evoking Procedures; Feedback by means of explaining; Think-time |  |
| Case 5  59% | Correct concept; No feedback after judgment  React to question teacher; Positive judgment ;  Conceptual understanding | React to spontaneous contribution; Feedback by means of explaining;  Spontaneous reactions; Fragmented concept; Neutral judgment; Conceptual understanding; Positive judgment | Feedback by means of follow-up question; Evoking Procedures; Evoking conceptual understanding; React to non-spontaneous contribution; Declarative knowledge |  |
| Case 6  55% | Declarative knowledge; React to non-spontaneous contribution; React to question teacher; Correct concept; Positive judgment  No feedback after judgment; Evoking declarative knowledge; Feedback by means of follow-up question | Non-complex thinking; Spontaneous reactions; Information, instruction or confirmation; No reaction to spontaneity; Neural judgment | Evoking conceptual understanding; React to spontaneous contribution; Feedback by means of follow-up question |  |
| Case 7  59% | React to question teacher; Non-complex thinking; No feedback after judgment; No reaction to spontaneity | Spontaneous reactions; Declarative knowledge; Fragmented concept  Procedures; Correct concept | Feedback by means of follow-up question; Evoking declarative knowledge; Think-time | React to non-spontaneous contribution; Evoking Procedures; Negative judgment; Information, instruction or confirmation |
| Case 8  58% | Feedback by means of follow-up question; Evoking conceptual understanding; Evoking declarative knowledge; React to question teacher; Incorrect concept | React to non-spontaneous contribution; Conceptual understanding; Fragmented concept; Correct concept; React to question teacher | Evoking Procedures; Correct concept; Procedures | Non-complex thinking; No feedback after judgment |
| Case 9  65% | Feedback by means of explaining; Neutral judgment; React to non-spontaneous contribution; Neutral judgment | Non-complex thinking; Evoking declarative knowledge; No reaction to pupils (non)concepts; No reaction to spontaneity; React to question teacher | Correct concept; Declarative knowledge; Positive judgment;  React to question teacher |  |
